# Architecture of *Anoteropora latirostris* (Bryozoa, Cheilostomata) and implications for their biomineralization

**DOI:** 10.1038/s41598-019-47848-4

**Published:** 2019-08-07

**Authors:** D. E. Jacob, B. Ruthensteiner, P. Trimby, H. Henry, S. O. Martha, J. Leitner, L. M. Otter, J. Scholz

**Affiliations:** 1 Department of Earth and Planetary Sciences, Macquarie University, North Ryde, NSW 2109 Australia; 2 Zoologische Staatssammlung München, Staatliche Naturwissenschaftliche Sammlung Bayerns, Münchhausenstraße 21, 81247 München, Germany; 3 Australian Centre for Microscopy and Microanalysis, The University of Sydney, Sydney, New South Wales 2006 Australia; 4 Present Address: Oxford Instruments Nanoanalysis, High Wycombe, UK; 5 Australian Research Council Centre of Excellence for Core to Crust Fluid System (CCFS)/GEMOC, Macquarie University, North Ryde, Australia; 6 Senckenberg Forschungsinstitute und Naturmuseen, Marine Evertebraten III, Senckenberganlage 25, Frankfurt, Germany; 7 Max Planck Institute for Chemistry, Particle Chemistry, Hahn-Meitner-Weg 1, Mainz, Germany

**Keywords:** Electron microscopy, Mineralogy

## Abstract

Cheilostome Bryozoa *Anoteropora latirostris, a* colonial marine invertebrate, constructs its skeleton from calcite and aragonite. This study presents firstly correlated multi-scale electron microscopy, micro-computed tomography, electron backscatter diffraction and NanoSIMS mapping. We show that all primary, coarse-grained platy calcitic lateral walls are covered by fine-grained fibrous aragonite. Vertical lateral walls separating autozooid chambers have aragonite only on their distal side. This type of asymmetric mineralization of lateral walls results from the vertical arrangement of the zooids at the growth margins of the colony and represents a type of biomineralization previously unknown in cheilostome bryozoans. NanoSIMS mapping across the aragonite-calcite interface indicates an organic layer between both mineral phases, likely representing an organic template for biomineralization of aragonite on the calcite layer. Analysis of crystallographic orientations show a moderately strong crystallographic preferred orientation (CPO) for calcite (7.4 times random orientation) and an overall weaker CPO for aragonite (2.4 times random orientation) with a high degree of twinning (45%) of the aragonite grains. The calculated Young’s modulus for the CPO map shows a weak mechanical direction perpendicular to the colony’s upper surface facilitating this organism’s strategy of clonal reproduction by fragmentation along the vertical zooid walls.

## Introduction

Bryozoans represent a diverse phylum of mostly marine, colonial suspension feeding animals, present in nearly 7,000 recent and about 20,000 fossil species in all types of marine habitats from the intertidal to the deep sea, and the polar regions towards the tropics. The phylum is unique in consisting exclusively of clonal animals. Colonies form by repeated budding of physically connected and intercommunicating modules called “zooids”^1^. Zooids are polymorphic and may be subdivided into ‘autozooids’, i.e. zooids responsible for feeding of the colony, and various ‘heterozooids’, which have specialised functions other than feeding^2^. Heterozooids include, for example, polymorphs responsible for active and passive defense (avicularia and spines), cleaning (vibracula) and colony stability (kenozooids). The modular construction allows two different levels of morphological analysis: that of the zooids and zooid chambers, and another of the budding and packaging of zooids to form various types of colonies^3^.

Bryozoan skeletal hard parts, consisting of calcite and/or aragonite^4,5^ are abundant throughout the geological record since the Ordovician^6^, which makes them interesting as potential paleo-environmental and -climatic proxy archives^7–10^, as well as ‘sentinels’ of ocean acidification^11–13^. Furthermore, they are an ideal group for the study of evolution, since they are abundant and morphologically complex. Most are sessile, calcify and laminarily encrust all types of substrata or grow erect. Death assemblages are commonly preserved *in situ* and reflect life assemblages^3^.

A prerequisite for evaluating and deciphering the environmental information potentially stored in bryozoan skeletal hard parts, however, is to better understand their skeletal architecture at the micro- to nano-scale and their biomineralization mechanisms^14^. In this study, we present a detailed characterization of the internal structure and biomineral architecture of colonies of recent *Anoteropora latirostris*, a bryozoan that is free-living and does not encrust with their basal colonial surface. The genus *Anoteropora* Canu & Bassler, 1927^15^ belongs to the suborder Cheilostomata which is the most diverse one of recent bryozoans, represented by about 5000 extant species^16^, and showing the highest developed polymorphism among all Bryozoa^17^.

**Figure 1:**
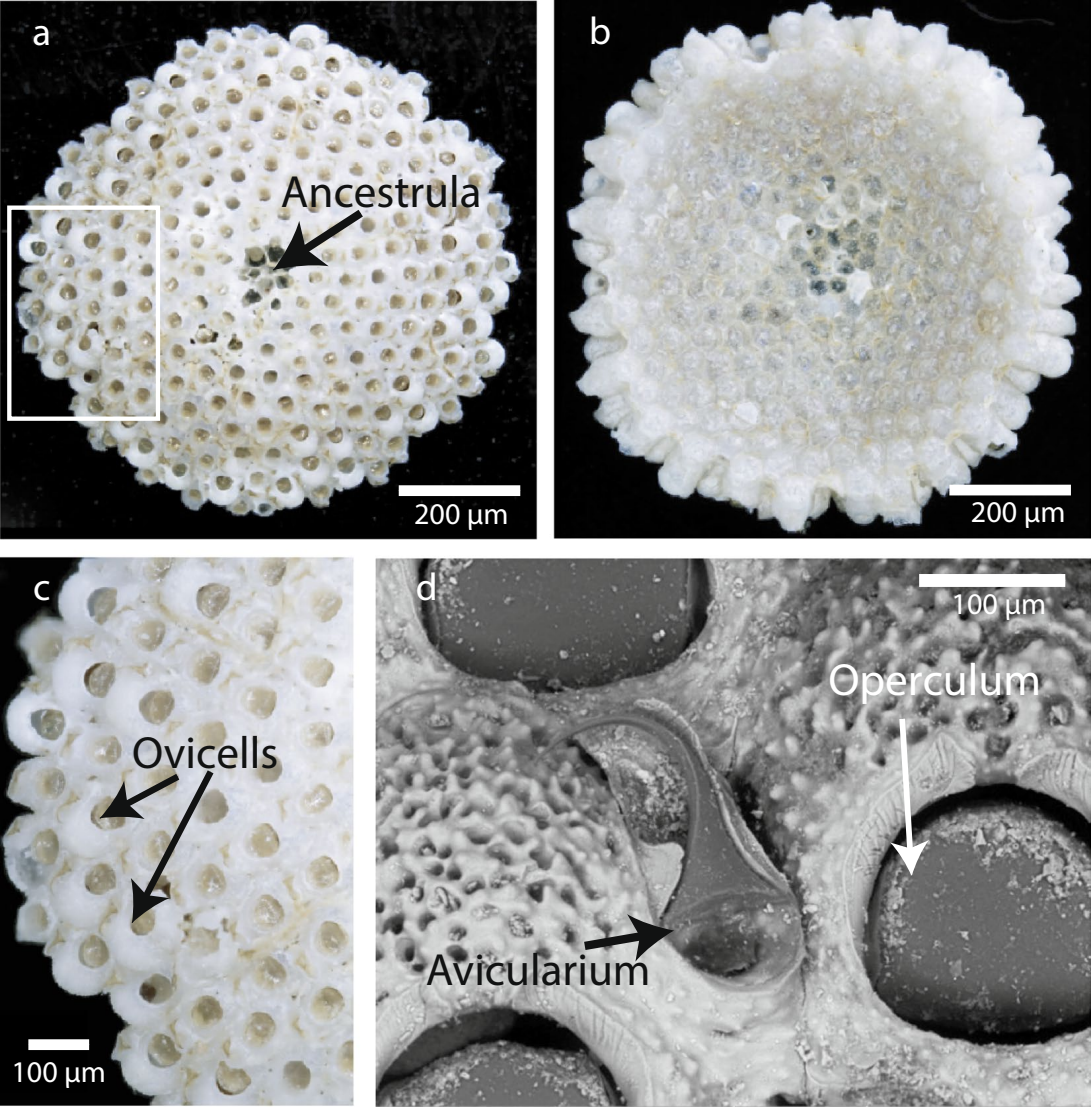
General aspects of *Anoteropora latirostris* Silén, 1947. **(a)** Apical (frontal) view of colony indicating the ancestrula (damaged in this individual). White rectangle shows the area of close-up in (c). **(b)** Basal view of colony. **(c)** Margin of colony with ovicellate autozooids. **(d)** SEM image of adventitious avicularium between two ovicellate autozooids, the orifices of which are closed by opercula. (**a**–**c**) SMF 60000; (d) SMF 60001 (see Supplementary Table 1).

Most of the marine bryozoans calcify and laminarily encrust all types of substrata or grow erect and attached with the base to a substratum. In contrast, some cheilostomes developed colonies that are not fixed to a substratum. While modern free-living bryozoans are motile and usually have cup-shaped colonies (lunulitiform) with the openings (orifices) in the autozooidal wall on the convex frontal (apical) surface similar to *Anoteropora latirostris* (Fig. 1a), the latter is not motile, but fixed to the substrate via rootlets^18^. Traditionally, studies on lunulitiform bryozoans have focused on free-living families, which, in terms of morphology, are ‘anascan’-grade cheilostomes lacking a frontal shield. Superficially very similar to free-living ‘anascans’, yet less well-studied, are ‘ascophoran’-grade lunulitiform bryozoans, such as *Anoteropora latirostris*. Colonies of *Anoteropora latirostris* are unilaminar, the autozooids are elongate and extend vertically from the frontal (apical) to the basal surface (Fig. 1b). Brood chambers (ovicells) (Fig. 1c) and avicularia (Fig. 1d) are present^19,20^.

## Results

### Colony morphology via X-ray Micro Computed Tomography (Micro-CT)

**Figure 2:**
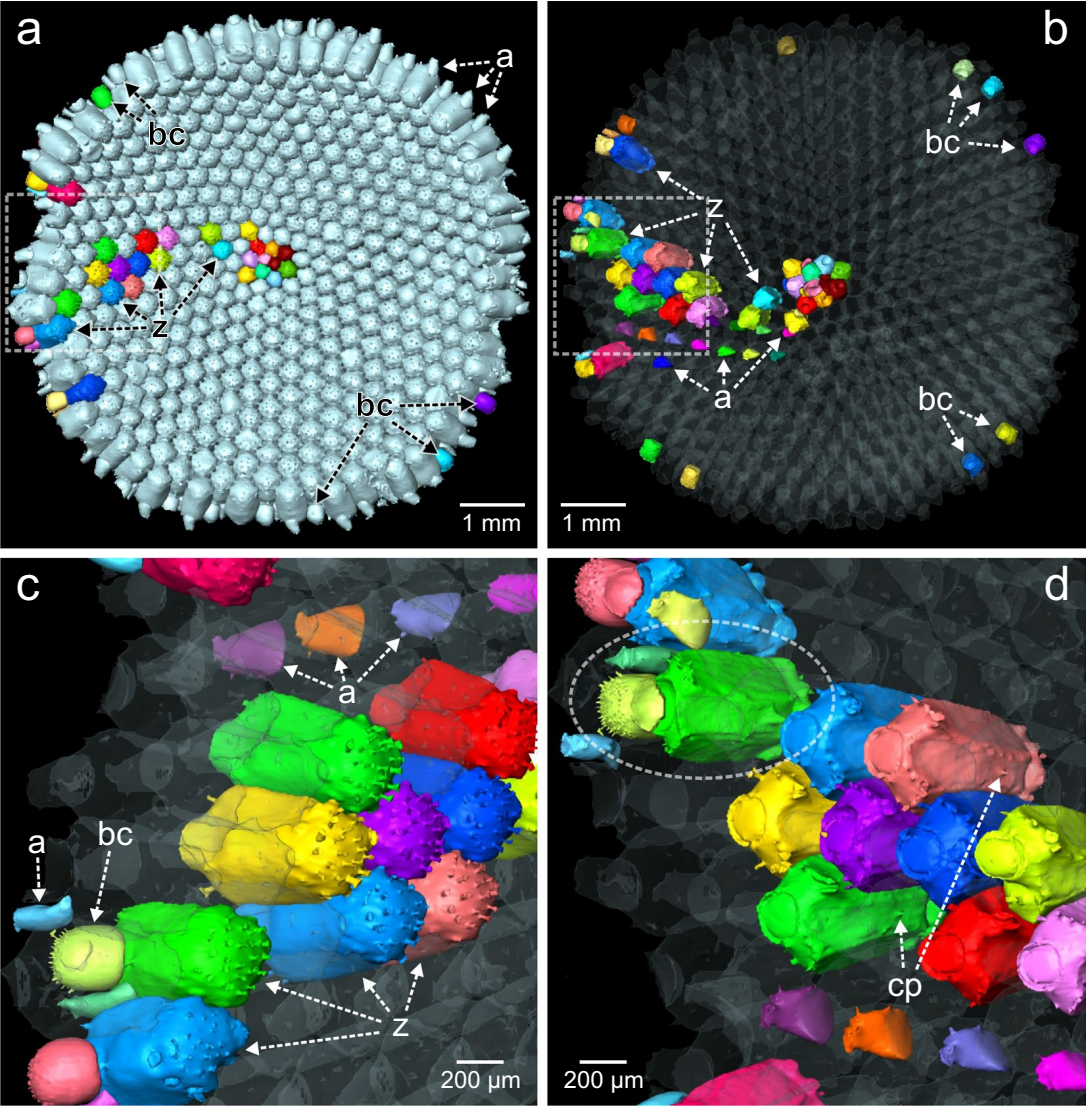
Endocasts from microCT of a colony of *Anoteropora latirostris* Silén, 1947 (sample SMF 60002 from station 283 KU); **(a)** Basal view of endocast with separated (coloured) zooid chambers. **(b–d)** Separated zooid chambers shown opaque and remaining endocast transparent. **(b)** Apical (frontal) view of endocast. **(c)** Basal view of marginal part of colony. **(d)** Apical (frontal) view of marginal part of colony; dashed rectangle in (b) outlines the enlarged areas shown in (c, d); dashed ellipse outlines the chamber of one autozooid and the brood cavity and avicularian chamber associated with the same autozooid. Abbreviations: a – avicularian chamber; bc – brood chamber; cp – connecting pore, z – autozooidal chamber.

Micro-CT images (including a 3D animation provided as Supplementary Video) allow accurate imaging of individual zooidal chambers and study of the zooid chamber and interconnection between zooids. Figure 2 shows the shape and general alignment of different zooid chambers and brood chambers in the colony with the endocast rendered transparent. Autozooidal chambers are block-shaped with rounded edges (marked as ‘z’ in Fig. 2), while avicularian chambers (marked as ‘a’) are smaller and flat ovoid-shaped. Brood chambers (marked as ‘bc’) are commonly present in autozooids at the distal edge of a colony (Fig. 1c). They are partly immersed, globular, and closed by the opercula. These opercula are clearly visible in most autozooids of the colonies studied (see also Supplementary Video), but are rarely preserved after death, thus indicating that the material used here is from live-collected and/or recently alive individuals. Connecting pores near the bottom of each autozooid (Fig. 2d) are used for interzooidal communication across the colony. Adventitious avicularia are connected with both the ‘host’ autozooid and the distally adjacent autozooid (Figs. 1d, 2c).

### Micron-scale architecture and mineral phase composition

**Figure 3:**
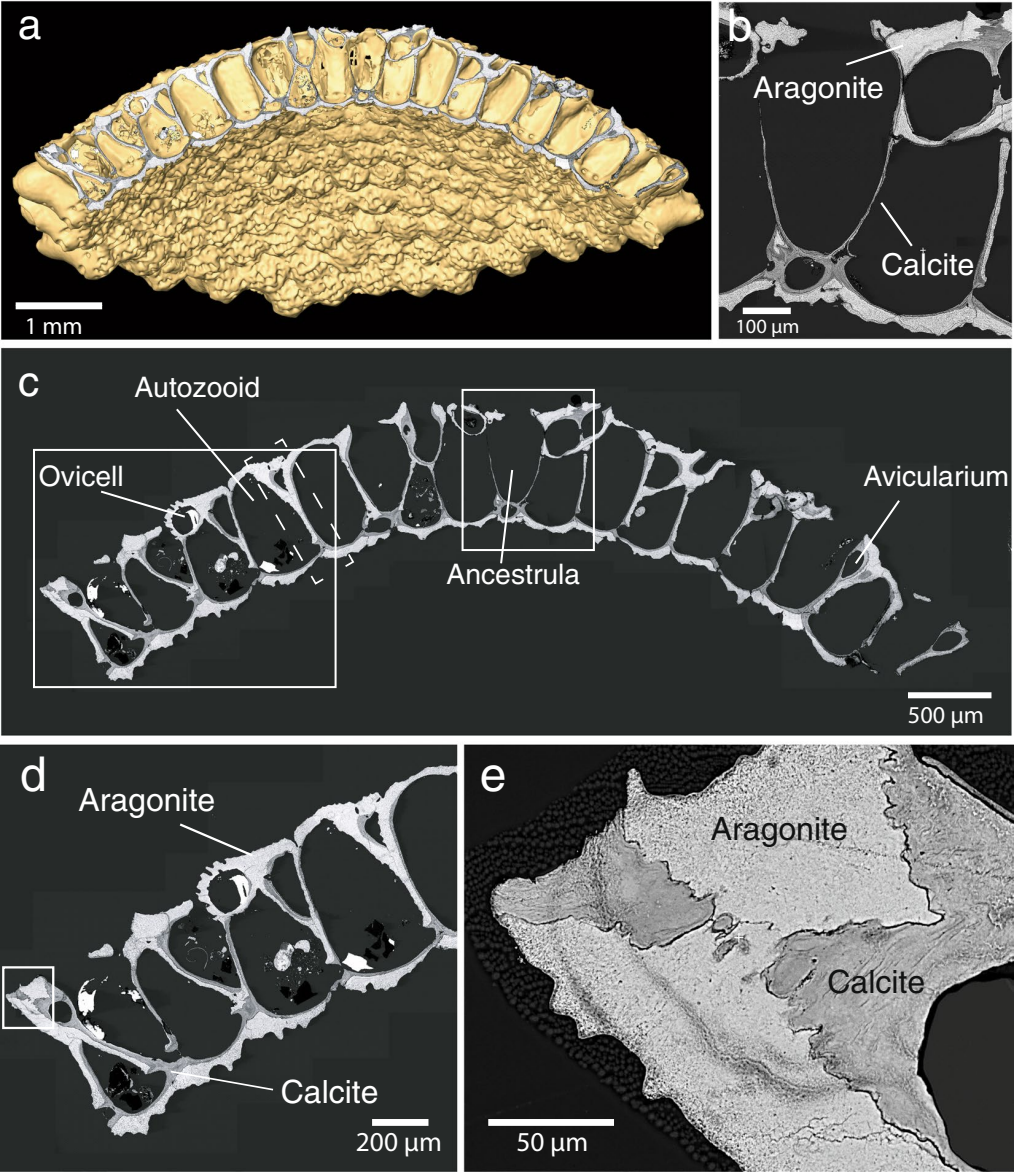
Mineralogy of *Anoteropora latirostris*. **(a)** Sectioned colony micro-CT overlain with BSE image of the polished cross-section shown in (c). **(b)** Close-up of ancestrula (central) area outlined by rectangle in **(c)** highlighting calcite in dark grey and aragonite in light grey. **(d)** Close-up of marginal area outlined by rectangle in (b). **(e)** Close-up BSE image of area outlined by rectangle in (d) showing calcite (dark grey) and aragonite (light grey) wall components. Dashed line in **(c)** outlines the area investigated by EBSD (Fig. 6).

Backscattered electron (BSE) imaging of colonies sectioned through their centre (Figs. 3a, S1, S2) show that most walls in the colony consist of two mineral phases distinguishable by their different greyscales (Fig. 3b–e), which are indicative of different compositions^21^. These phases were identified as calcite (darker grey) and aragonite (light grey) via Electron Backscatter Diffraction (EBSD) analysis.

**Figure 4:**
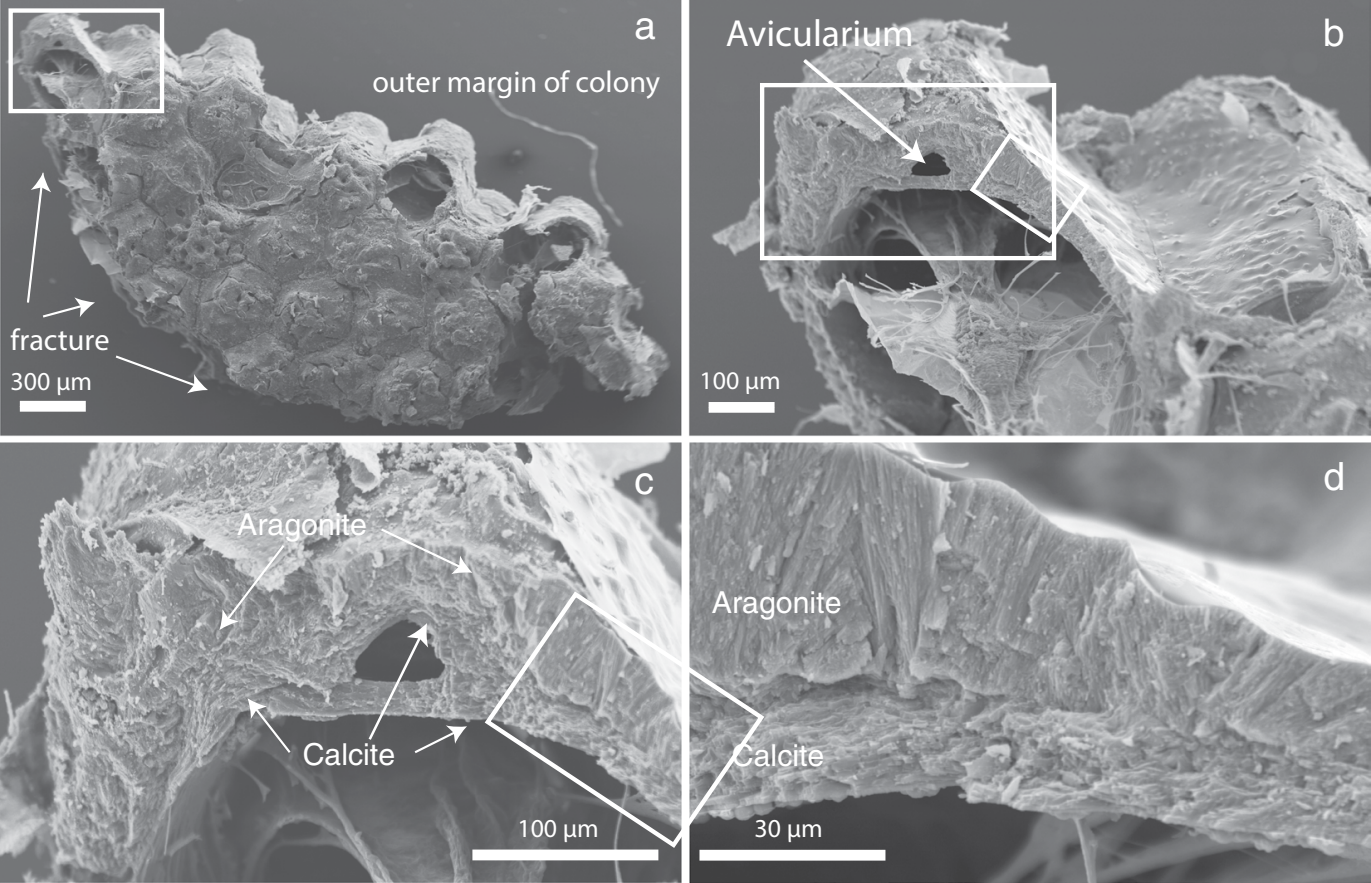
SEM images of a skeleton of *Anoteropora latirostris* (sample SMF 60004). **(a)** Basal view of broken marginal piece of colony. **(b)** Fractured cross-section through autozooid near the basal wall (close-up of area marked with a rectangle in (a)) showing a fracture through three of the six walls defining the autozooid chamber. Outer marginal wall contains an avicularium; parietal and retractor muscles traverse the autozooid chamber. White rectangles indicate close-up areas shown in (c) - large rectangle and (d) – small rectangle. **(c)** Marginal wall shows platy calcite on the inside and enclosing the avicularium (cf. Fig. 3c) and fibrous aragonite on the outside. **(d)** Close-up of the lateral wall area marked with a rectangle in (c) and (b) consisting of a thick layer of fibrous aragonite and a thin layer of platy calcite towards the autozooid chamber.

**Figure 5:**
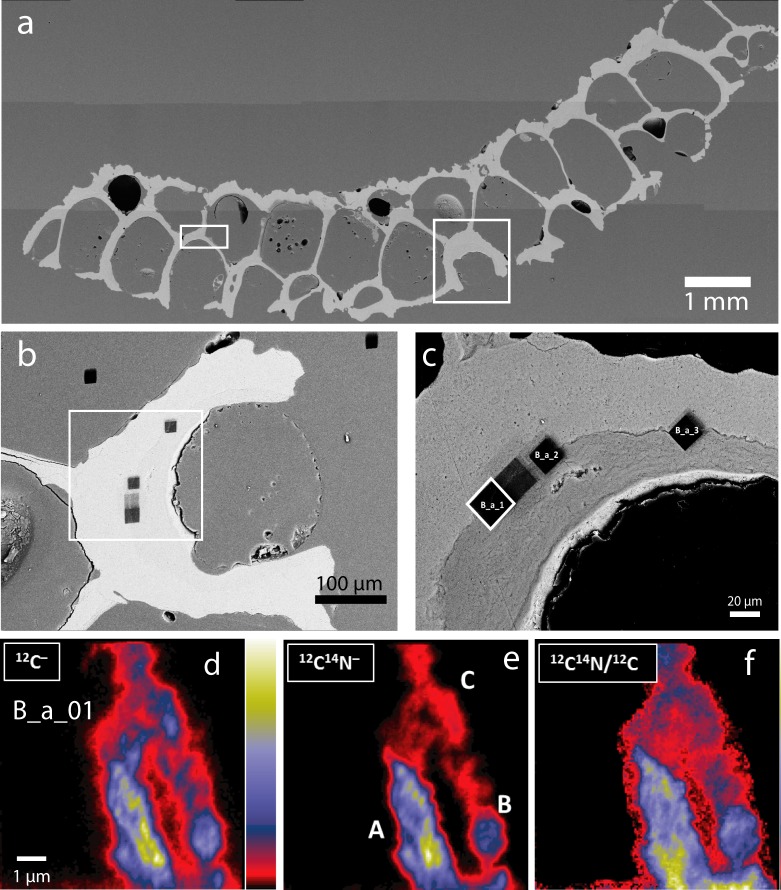
SEM images and NanoSIMS maps of sectioned and polished epoxy embedded *Anoteropora latirostris* colony. **(a**–**c)** Show the areas mapped by NanoSIMS after analyses at the interface between calcite (dark grey in **c**) and aragonite (light grey in c). Two maps in the epoxy resin were measured for comparison (see Table S2). **(d–f)** NanoSIMS maps for area outlined by white rectangle in (c): **(d)** Carbon concentrations (measured as ^12^C^−^), **(e)** nitrogen concentrations (measured as ^12^C^14^N^−^) and (f) C/N ratios show higher concentrations along the interface between aragonite and calcite than on either side of it (compare colour scale to the right of d), indicating the presence of an organic membrane along the interface. NanoSIMS maps are each 15 × 15 µm^2^, for quantitative data see Table S2.

In detail, the different units of each mineralized zooid chamber consist of a specific combination of aragonite and calcite as verified through SEM study of sixteen different colonies (Supplementary Table S1): judging from the sections we studied, the outside layer of the wall of the colony consists of fibrous aragonite (Figs. 3c, 4, Supplementary Figs. S2, S3). The inner layer of the basal and (most) apical walls, which face the zooid, consist of platy calcite (Figs. 3c, 4b, Supplementary Figs. S2, S3). All vertically-oriented interzooidal walls consist, in regard to the centre of the colony, of a distal layer of aragonite and a proximate layer of platy calcite. All walls are therefore bimineralic and, in addition to calcite, contain a secondary fibrous aragonite layer. The calcitic walls are generally thinner than the aragonite walls (see Supplementary Fig. S1 for measurements in one of the colonies: calcite layer − 3–35 µm in thickness, 5–45 µm for aragonitic walls, occasionally up to 80 µm). Boundaries between calcitic and aragonitic walls are irregular and can be crenulated (Fig. 3e). To investigate whether an organic membrane separates these two mineral phases, we employed NanoSIMS mapping. This method combines the advantage of submicron spatial resolution as is required here, with minimal additional sample preparation, thus reducing the risk of artefacts. Carbon and nitrogen distribution maps by NanoSIMS analysis across the calcite-aragonite interface (Fig. 5a–c) show significantly increased signals for these elements along the interface (Fig. 5d–f, Supplementary Fig. S4), which is a clear indication for the presence of a thin organic layer between calcite and aragonite.

The interzooidal walls at the apex of the colony, representing the central area including the ancestrula and thus the oldest part of the colony, are damaged or missing in many specimens (Fig. 1a, Supplementary Fig. S2). However, in colonies where these are preserved, the walls delineating the ancestrula are purely calcitic and lack the aragonite layer (Fig. 3c), which contrasts with the interzooidal walls of subsequent generations of colony modules (radially away from the centre) in the same samples.

### Crystallographic orientation of calcite and aragonite grains via Electron Backscatter Diffraction (EBSD)

**Figure 6:**
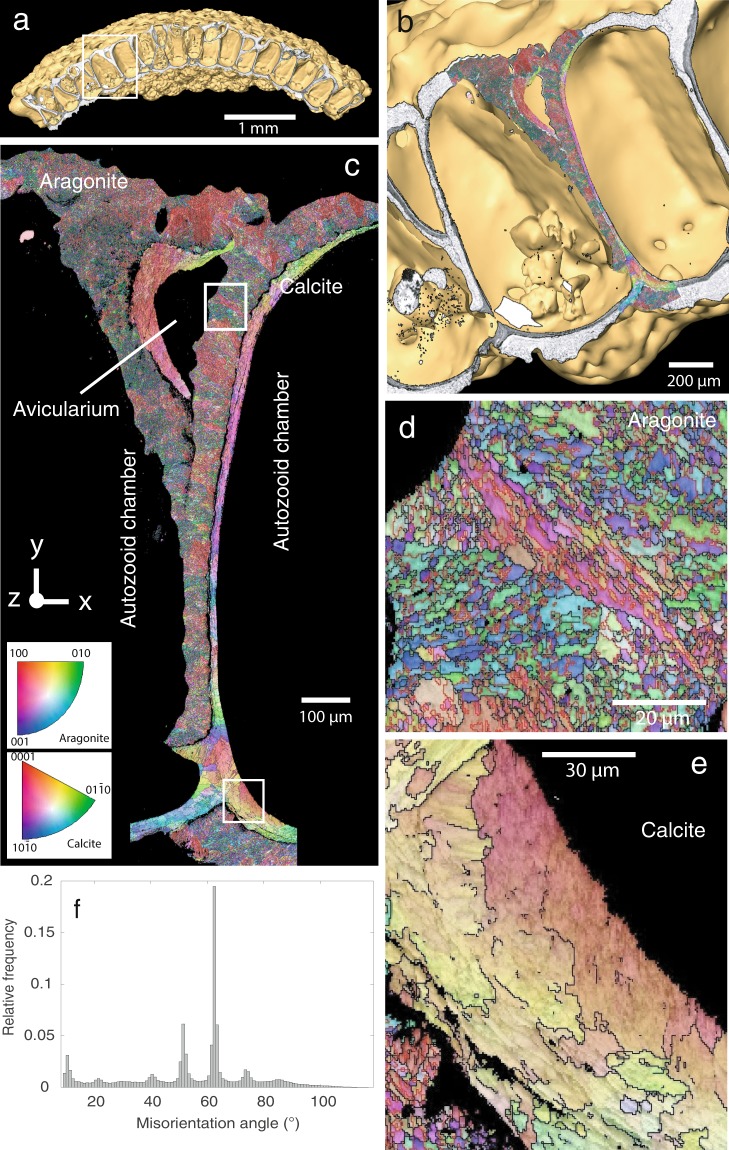
Crystallographic preferred orientations in the skeleton of *Anoteropora latirostris*. **(a)** Sectioned colony micro-CT overlain with BSE image of the polished cross-section shown in Fig. 3c. **(b)** Close-up of area outlined by rectangle in (a) overlain with crystallographic orientation map from Electron backscatter diffraction analysis (EBSD). **(c)** Orientation map of a lateral vertical wall segment of the colony (see also Supplementary Fig. S2 for high resolution map). **(d)** close-up of an aragonitic segment outlined by rectangle in (c). Red grain boundaries are twin boundaries, colours as in the scale in (c). **(e)** close-up of a calcitic segment outlined by rectangle in (c). **(f)** Histogram of misorientation angles between aragonitic grains in (c) show pronounced maxima for the main twinning angles in aragonite at around 63° and 52**°**.

An entire transect of the sectioned and polished colony was analysed by EBSD (Fig. 3c: white dashed rectangle, Fig. 6b and Supplementary Video). The resulting crystallographic orientation map for aragonite and calcite grains in Fig. 6b,c and Supplementary Fig. S6 is coloured according to the inverse pole figure colour schemes for aragonite and calcite (insets), assuming the reader’s perspective. For aragonite and for calcite, areas in red have their crystallographic c-axis pointing north or south in the map. Green denotes the a-axis in aragonite and the calcite [01¯10] axis, while blue is the aragonite b-axis, and the [10¯10] axis in calcite, respectively. Intermediate colours are crystallographic orientations intermediate to these three.

The maps allow grain size analysis for both mineral phases and show that these differ significantly between calcite and aragonite in the colony walls: While calcite grains are relatively large (Fig. 6e), mostly around 600 µm, occasionally up to 1400 µm, aragonite grains are predominantly less than 100 µm in size (Fig. 6d, Supplementary Fig. S7). Twinning was not observed in calcite, while a significant proportion of aragonite grains (i.e. 45%) are twinned with a misorientation angle of 64° (Fig. 6f), which is close to the angle of twinning on {110} in aragonite. Aragonite shows a predominantly small-grained fraction (~75 µm^2^). Calcite grain sizes are distributed over a large range between ~50 µm^2^ and ~1,3 mm^2^ with no significant maximum (Supplementary Fig. S7).

**Figure 7:**
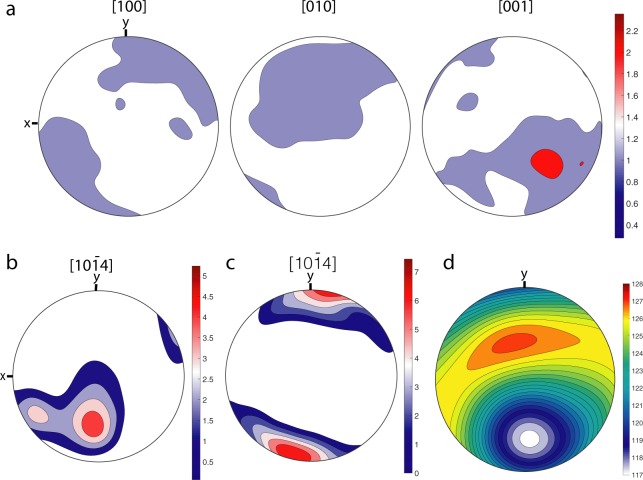
Pole figures for aragonite and calcite in the skeleton of *Anoteropora latirostris* Silén, 1947. **(a)** pole figure for aragonite crystals across the entire orientation map in Fig. 6c showing the orientation of the prism axis (N = 1,037,343). **(b)** calcite orientations in the avicularium wall (see Fig. 6c) and **(c)** across the entire orientation map in Fig. 6c. **(d)** Young’s modulus (in GPa) for the entire orientation map.

Calcite and, to a lesser extent, aragonite show significant intra-grain misorientation as indicated by the changing colours in Fig. 6c–e. This is a typical feature in biominerals and an effect of the particulate nature of the grains^22^, sometimes referred to as ‘mesocrystals’^23,24^. Aragonite crystals show a weaker crystal preferred orientation (Fig. 7a) than calcite crystals (Fig. 7a,b).

The growth face of any calcite crystal is usually the face of the cleavage rhombohedron, normal to the [10¯14] axis and with a constant angle of 44° to the calcite c-axis. The pole figures for calcite across the complete orientation map show a relatively strong alignment (five multiples of uniform distribution) of the [10¯14] axis (Fig. 7b). In the wall of the avicularium (Fig. 1d), the calcite [10¯14] axis is oriented sub-orthogonal to the surface of the colony (close to the y-direction; mostly red colours in Fig. 6c) and has a stronger alignment (seven multiples; Fig. 7c) compared to that of the overall orientation map for the transect. The orientation of the calcite crystals along the entire vertical interzooidal wall changes gradually from an alignment of the c-axis close to the y-axis (orthogonal to the surface of the colony, Fig. 6a, b) at the surface and the underside of the colony (mostly green colours in Fig. 6c) via an orientation pointing more to the reader (z–direction, mostly red-purple colours in Fig. 6c).

## Discussion

### Bimineralic bryozoan skeletons

Little is known about the ultrastructure and the biomineralization processes of bryozoan skeletons compared to the sizeable body of work available on other phyla such as molluscs, cnidarians or echinoderms. Cheilostome bryozoans are known since the Late Jurassic and originally had monomineralic calcitic skeletons. Their mineralogy diversified with many cheilostome clades convergently evolving aragonitic or bimineralic calcitic-aragonitic skeletons by the Late Cretaceous^25^. In bimineralic species, the aragonitic parts typically comprise the outer surfaces of the colony walls^5,21^ and/or the frontal shield. This is similar for *A. latirostris*, where an aragonite layer covers the entire surface of the colony (Fig. 3c). In addition to this, in this species we observe aragonite layers also along the lateral walls of each zooidal chamber (Figs. 3, 4, Supplementary Figs S2, S3). Taking into account the general growth direction of the colony, namely from the centre (apex) radially to the margins, the aragonitic layers on the vertical interzooidal walls are precipitated distally (Fig. 3b–d), thereby resulting in the colony being radial symmetrical whereas the zooids are not. We found these zooid walls with an aragonite layer covering the calcite wall on the distal side of the zooid chamber in all *A. latirostris* colonies that we studied (see Table S1 and Methods for a full list of studied specimens) and interpret them as primary and integral parts of the *A. latirostris* skeleton. We consider it unlikely that this feature could be a result of diagenesis, because all colonies show intact opercula (Fig. 1d), indicating that they were alive or recently alive at the time of sampling. Furthermore, the aragonite layer composes one side of the wall to its full height and with relatively constant thickness, which is typical for a regular architectural element of the skeleton, whereas diagenetic aragonite could be expected to form more random morphologies and cavity fillings.

### Ultrastructure and mechanical properties

Ultrastructures in bimineralic bryozoan skeletons usually differ according to their mineralogy^25–27^ and the same is observed for *A. latirostris* here: Calcitic structures have platy or lamellar morphology, while aragonites show a fibrous fabric (Fig. 4b,c). Unlike calcite, aragonite grains are most often crystallographically twinned (Fig. 6d), which is a common observation for aragonites in biominerals^22,28^.

Unlike the shells of some molluscs, where several hierarchical orders can be identified^29,30^ the *A. latirostris* skeleton does not show a pronounced hierarchy of distinct architectural units. Aragonite fibres and calcite platelets show a crystallographic preferred orientation (CPO), but not as strong as in bivalve shells: Areas of similar colouration in the orientation map (Fig. 6c) show domains of crystallites with very similar orientation for both minerals. Pole figures (Fig. 7) show a relatively strong preferred orientation for the calcitic crystallographic [10¯14] axis of up to 7.4 times uniform (uniform equals random orientation) (Fig. 7a,b) and a weaker preferred orientation for [001] of aragonite of 2.4 times uniform. Compared to the textural strength of bivalve shells, which can have textural strengths of 12.5 times uniform^22,31^, these maxima are weak in *A. latirostris*.

In mollusc shells the strongly controlled CPO across the entire shell maximizes their mechanical properties by creating a plane of (quasi-) isotropy of the Young’s modulus for the otherwise anisotropic crystals, thus maximizing the stiffness coefficients in all directions across this plane in the shell^22,31,32^. In aragonitic mollusc shells, this mechanical effect is further enhanced by a spread of the crystal orientation angle due to extensive twinning. Aragonite grains in the *A. latirostris* skeleton show a high degree of twinning (45% of all grains in Fig. 6c have at least one twin boundary), but the textural strength of aragonite (2.4 times uniform; Fig. 7a) and calcite (5–7.4 times uniform; Fig. 7b,c) taken individually is much weaker compared to the bivalve *T. derasa* (12.5 times uniform^22^). Nevertheless, the combination of the two mineralogically different layers in the skeleton results in a similar, even slightly higher range of values of 117 to 126.5 GPa for the Young’s modulus (compared to 80 to 106 GPa in *T. derasa*^22^). The anisotropy of the Young’s modulus for the *A. latirostris* skeleton results in a value of 7.8%, thus, it is significantly more isotropic in all directions than for the shell of *T. derasa* (28%^22^).

A noteworthy and significant difference between the Young’s modulus for shells of the bivalve *T. derasa* and the skeleton of the bryozoan *A. latirostris*, however, is that the stiffest direction in the further is perpendicular to the shell’s dorsal surface (perpendicular to the growth lines in *T. derasa*^22^), whereas this direction (i.e. perpendicular to the colony’s apical surface) is the weakest in *A. latirostris*. It could be speculated that this difference in mechanical properties likely supports the function of the mineralized parts of these organisms: Molluscs most likely developed their architectural design aiming at optimizing mechanical isotropy as a strategy in resistance to predators that may have provided a significant evolutionary advantage. *A. latirostris* colonies, on the other hand, proliferate by fragmentation of the colony along the interior vertical walls between zooids^33^ and maintaining the weak mechanical direction perpendicular to the colony’s apical surface in this species is potentially a strategy to facilitate this fragmentation process.

#### Insights into the biomineralization processes

We observed that the spatial distribution pattern of aragonite coating on calcite walls, and the construction of the walls follows a strikingly symmetrical pattern on colonial and on zooid level with only the distal side of the vertical zooidal walls layered with aragonite (Fig. 3). The regular arrangement and thickness of the aragonite layers and the freshness of the sample material excludes a diagenetic origin and therefore raises the question about how these walls are formed.

A unifying principle of biomineralization is that the minerals nucleate and grow with the support of an organic template, which promotes biomineralization by minimizing surface energies and provides binding sites for calcium^34^. The central organic cuticular layer^35^ observed in compound walls in bryozoan skeletons is thought to act as such a biomineralization template, but similar organic cuticles have never been described between calcite and aragonite layers in walls. NanoSIMS distribution maps for carbon and nitrogen across the calcite-aragonite interface (Fig. 5a,b) show significantly increased signals for these elements along the interface (Fig. 5c–e, Supplementary Fig. S4), which is a clear indication for the presence of a thin organic layer between calcite and aragonite. This strongly suggests that the general principles of biomineralization as observed in marine invertebrates, such as nucleation and growth of crystals involving an organic template^34^, may also hold for bryozoans.

Macroscopically, colonial growth of *Anoteropora latirostris* proceeds radially, starting from the centrally located ancestrular zooid (Fig. 1a), which is also the only zooid composed solely of calcite (Fig. 3c). This suggests that calcite is the primary CaCO_3_ phase, which is secreted from the epithelial cells of the hypostegal coelom of the zooid in each of the zooid chambers. Most of the remaining skeleton, however, is aragonitic: Aragonite makes up the outer layer of the extrazooidal basal walls, of the apical walls as well as most of the distal parts of the lateral walls (Figs. 3, 4, Supplementary Figs. S3, S4). This suggests that aragonite is a secondary phase, secreted from the cells of a tissue layer covering the exterior of the colony in its entirety (i.e. frontal, basal walls and lateral margins of the colony).

The asymmetric distribution of aragonite only at the distal side of each autozooid chamber (Fig. 3) can be understood by studying the radial growth mechanism of the colony: In a living colony, growth of the colony occurs via budding and internal separating of a single autozooid into two individuals^3^. Thereby, the distal lateral vertical walls of each newly-formed autozooid chamber comprise the margin of the growing colony and are therefore in direct contact with the exterior tissue that covers the entire colony. Hence, we conclude from our observations that *A. latirostris* possesses two different types of calcifying tissue, one being the epithelial tissue of the autozooidal hypostegal coelom, which produces calcite and the other being the tissue covering the colony which produces aragonite. Such cellular differentiation, as suggested here, would follow observations made in bivalves, where the regulation of which polymorph of calcium carbonate - aragonite or calcite - forms during shell growth is achieved by cellular differentiation^36^, and cell secretion plasticity is retained to accommodate shell repair processes^37^.

This bimineralic calcification pattern is significantly different to what has been documented for other bimineralic bryozoan species so far. Typically, secondary aragonitic layers are limited to the frontal shields in bimineralic colonies, such as *Pentapora fascialis*^21^, but do not comprise the vertical interzooidal walls. The difference in growth form between this species and *A. latirostris* is that the latter has a lunulitifom (cup-shaped) mode of growth, and the colony only consists of one zooid layer. Budding at the margins of the cup-shaped colony exposes the distal sides of newly formed calcitic interzooidal walls to the aragonite-forming exterior tissue, unlike in *Pentapora fascialis* where only the frontal surfaces (the frontal shields) for the bilateral two-zooid-layer colony are exposed for aragonite formation.

## Methods

### Systematic account

The study object, *Anoteropora latirostris* Silén, 1947^38^, is a cheilostome bryozoan of the family Mamilloporidae Canu & Bassler, 1927 that has cup-shaped colonies anchored by rhizoids (rootlets) that emanate from the concave basal surface. Autozooids (feeding zooids) are hexagonal in outline and arranged in rows radiating from the centrally located ancestrula (initial zooid, Fig. 1a,b). *A. latirostris* has a calcified frontal shield with occasional marginal pores and with a large central circular opening (orifice, Fig. 1d) for passage of the lophophore and tentacles. One avicularium (specialized defense zooid) is positioned on the surface of each autozooid (Fig. 1d) and has an acute, outwardly curved distal end. In infertile autozooids, the avicularium is located distolateral or distal of the orifice, while in fertile autozooids, it occurs lateral of the brood chamber (ovicell, Fig. 1c). The single avicularium occurring both in fertile and infertile autozooids allows unequivocal identification of the species as *A. latirostris*. *A. latirostris* is widespread in the Indian Ocean from the Cape of Good Hope to the southern Red Sea and the Malacca Strait^20^.

#### Collection and repository of material

In total 240 colonies of the motile bryozoan species *Anoteropora latirostris* Silén, 1947 have been used for this study (Table S1). 239 of those were studied by light microscope and 16 specimens were chosen for SEM study. Micro-CT was carried out on 2 samples and one each was chosen for NaonSIMS and EBSD. The material was collected in March 1987 during RV *Meteor* expedition 5/2 “Mindik” from dredged sediment in the Gulf of Aden^39^. All material is from stations 236 KD (12° 21′24.01″N; 43° 26′53.99″E) and 283 KU (12° 30′54″N, 44° 47′42″E). Both stations represent shallow banks of 35–45 m (236 KD) and 70 m (283 KU) depth and the sea bed was covered with coarse carbonate sands and gravels, shells and coral debris. This type of shallow water sediments was not sampled elsewhere during this expedition, as the main goal was to explore the deep sea habitats. See Supplementary Fig. S8 and Supplementary Methods for details on sampling locations and collection. The sample material was preserved in 4% formalin onboard and were watered and transferred to 70% ethanol upon arrival in Frankfurt, and later dried. No further sample preparation other than breaking or cutting of the colonies was carried out prior to analysis. All samples are stored at the Senckenberg Natural History Museum (SNG) in Frankfurt am Main (SMF 60000–60019) and the Bavarian State Collection of Zoology in Munich (ZSM20190252-ZSM20190253).

#### Sample preparation, light and electron microscopy

A Leica MZ12.5 stereomicroscope with a maximum magnification of 120-x was used for choosing suitable colony fragments for SEM imaging.

SEM images of uncoated fragments were produced using a Tescan Vega 3 scanning electron microscope equipped with a low-vacuum chamber at the Senckenberg am Meer (SMW) in Wilhelmshaven, Germany (Fig. 1d). Specimens imaged at the SGN (Fig. 4, Supplementary Fig. S3) were coated in gold/palladium (20/80) and using a Camscan CS 24 SEM producing secondary-electron images. BSE images of sectioned and polished colonies were obtained using a JEOL JXA 8200 electron probe microanalyzer (EPMA) at the University of Mainz, Germany. Specimens were imaged using 15 kV acceleration voltage, 8 nA beam current at a working distance of 11 mm. BSE images were merged using Photoshop CS5 and skeleton thickness was measured using ImageJ. For NanoSIMS mapping, colonies were embedded in epoxy resin, sectioned and polished at Max-Planck Institute for Chemistry, Mainz, Germany following routine methods.

#### X-ray micro-CT

X-ray micro-computed tomography (micro-CT) scans were produced using a Phoenix nanotom m (GE Measurement & Control, Wunstorf, Germany). One specimen was scanned at a voltage of 120 kV and a current of 80 μA for 1600 projections over 53 minutes. Two specimens previously used for EBSD analysis (see below) were scanned at a voltage of 130 kV and a current of 90 μA for 1440 projections over 48 minutes.

Data sets (voxel size 4.44 µm for the untreated specimen, 3.04 µm for the EBSD treated specimens) were further processed using Amira 6.4 (Thermo Fisher Scientific, Hillsboro, Oregon, USA). Surface models were produced by threshold segmentation. Endocasts of zooid chambers were segmented semi-manually. For the Supplementary video the EBSD section was co-registered with the 3D-CT data set largely following the procedure outlined by Handschuh *et al*.^40^.

#### Electron backscatter diffraction

EBSD was carried out on one sectioned and polished colony of *A. latirostris* using a Zeiss Ultra Plus field emission gun scanning electron microscopy (FEG SEM) equipped with an Oxford Instruments Nordlys Nano EBSD detector. Crystallographic preferred orientation (CPO) maps were acquired using the AZtec software v3.0 and were processed using the Channel5 data processing software. Diffraction patterns were collected using an accelerating voltage of 15 kV, a beam current of ~10 nA, a detector resolution of 336 × 256 pixels and at a rate of 40 patterns per second.

In total, 7 separate maps were combined to form a transect across the full colony width, with a measurement spacing of 200 nm. The indexing rate across the full area was typically 90–95% in the areas without significant porosity.

Grain sizes as observed by TEM are usually in the nanometre range in biominerals^41,42^, hence the chosen ‘grain’ size cut-off for EBSD defines domains of several crystallographically well-aligned nanograins, rather than individual aragonite grains and depends on the spatial resolution of the method.

EBSD data were processed using the MTex toolbox for Matlab^43,44^. Measurement bearing a mean angular deviation (MAD) value greater than 1.3 were discarded. Grains were computed using a 10° cut-off angle. Grains smaller than 10 pixels were discarded to prevent bias caused by potential indexing errors. The remaining grains were then smoothed and missing data within grains were interpolated.

Data are plotted in pole figures (Fig. 7a–c), which are stereograms with axes defined by an external reference frame using the direction tangential (X) and perpendicular to the convex surface of the colony (Y) and the axis normal to the X-Y plane (Z). Accumulation of points around a specific direction in the pole figures (pole maxima) shows a degree of texture in the polycrystalline material, quantified according to the colour scales in the figures. For calcite (N_grains_ = 556), an orientation density function (ODF) with a bandwidth of 10° was calculated using the mean orientation of each grain (i.e. one point per grain pole figure). For aragonite (N_grains_ = 11,389), as twinning is occurring, all EBSD measurements were considered for the ODF calculation (N_measurements_ = 1,037,343; bandwidth = 10°). The resulting ODFs were then plotted on a lower hemisphere, equal area projection. Contours represent multiples of a uniform distribution.

The twinning percentage was defined as 100*((N-N_t_)/N) with N the number of grains computed without taking twinning into account and N_t_ the number of grains computed by taking twinning into account. Twin boundaries for aragonite were defined as a 63.8° + /−5° rotation around the [001] axis. The strength of the crystallographic preferred orientation is usually estimated using the J- index^45^ and the M-index^46^. For a fabric with random (strong) crystallographic orientation, the J-index will approach one (infinity), while the M –index trends towards zero (one). Here, we determined a J-index of 11.8 for calcite (1.23 for aragonite) and an M-index of 0.48 (0.02 for aragonite).

Orientation maps (Fig. 6c) are plotted using the Channel5 software and are coloured according to the inverse pole figure colour schemes for aragonite and calcite (insets), showing the crystallographic direction parallel to the map’s Y (vertical) direction. For orthorhombic aragonite, areas in red have their crystallographic [001] axis pointing up or down the map, green denotes the [100] axis and blue the [010] axis. For rhombohedral calcite, areas in red have their crystallographic [0001] axis pointing up (North) or down (South) in the map, green denotes the [01¯10] axis and blue the [10¯10] axis.

The Young’s modulus in Fig. 7d was calculated using the Voigt-Reuss averaging scheme for the aggregate elastic constant with the single crystal elastic constants for calcite^47^ and for aragonite^48^. The maximum anisotropy for the Young’s modulus was calculated as 200*(Ym_max_ − Ym_min_)/(Ym_max_ + Ym_min_) with YM_max_ and YM_min_ the maximum and minimal values (in GPa), respectively.

#### NanoSIMS analysis

NanoSIMS analyses in this study were carried out with the NanoSIMS 50 ion probe^49^ at the Max-Planck-Institute for Chemistry, Mainz, Germany. The Cameca NanoSIMS 50 ion probe is a secondary ion mass spectrometer designed for high spatial resolution isotope measurements with high sensitivity and simultaneous detection of up to 5 (NanoSIMS 50) isotopes. All measurements were performed in “ion imaging” mode, by rastering a primary Cs^+^-beam (100 nm diameter, ~1 pA) over several regions of interest. Prior to analysis, selected sample areas were pre-sputtered with a high current primary beam (~20 pA) to remove the gold coating. Secondary ion images of ^12^C^–^, ^12^C^14^N^–^, and ^28^Si^–^ from selected sample areas (10 × 10 µm² and 15 × 15 µm², respectively) were acquired in multi-collection mode.

Two to three image planes (256 × 256 pixels) were recorded for each analysis area with integration times of 10 ms/pixel each. The ion counting rates were corrected for quasi-simultaneous arrivals (QSA) with correction factors according to Slodzian *et al*.^50^ and Hillion *et al*.^51^ Corrections were applied individually for each region of interest. For data reduction and processing, in-house software developed at the MPIC was used. Since the sample was embedded in epoxy resin, care was taken to identify potential contamination from the epoxy on the secondary ion images of ^12^C^−^ and ^12^C^14^N^−^. For this, two locations in the epoxy (Table S2), away from the sample, were measured and ^12^C^14^N^−^/^12^C^−^ ratios obtained there were compared with those on the sample to monitor potential contamination of the sample area with epoxy resin. One of the seven areas measured (B_a_01#C) was identified as contaminated in this way and rejected (Table S2).

## Supplementary information

Supplementary Info

Supplementary Video 1

Dataset 1

## Data Availability

All data is available with this ms, the Supplementary Material or from the corresponding author on request.
