# Supplementary material for: Architecture of Anoteropora latirostris (Bryozoa, Cheilostomata) and implications for their biomineralization
Source: Sci Rep. 2019 Aug 7;9:11439. doi: 10.1038/s41598-019-47848-4 (PMC6685955; doi:10.1038/s41598-019-47848-4)
Supplement: Supplementary file 1 — Supplementary Info [file 41598_2019_47848_MOESM1_ESM.docx]

**Supplementary Information to:**

Architecture of *Anoteropora latirostris* (Bryozoa, Cheilostomata) and implications for their biomineralization

D.E. Jacob^1*^, B. Ruthensteiner^2^, P. Trimby^3,4^, H. Henry^1,5^, S.O. Martha^6^, J. Leitner^7^, L.M. Otter^1^, J. Scholz^6^

^1^Department of Earth and Planetary Sciences, Macquarie University, North Ryde NSW 2109 Australia

^2^Zoologische Staatssammlung München, Staatliche Naturwissenschaftliche Sammlung Bayerns, Münchhausenstraße 21, 81247 München, Germany

^3^Australian Centre for Microscopy and Microanalysis, The University of Sydney, Sydney, New South Wales 2006, Australia

^4^present address: Oxford Instruments Nanoanalysis, High Wycombe, UK

^5^Australian Research Council Centre of Excellence for Core to Crust Fluid System (CCFS)/GEMOC, Macquarie University, North Ryde, Australia.

^6^Senckenberg Forschungsinstitute und Naturmuseen, Marine Evertebrates III, Senckenberganlage 25, Frankfurt, Germany

^7^Max Planck Institute for Chemistry, Particle Chemistry, Saarstrasse 23, Mainz, Germany

**Supplementary Table S1.** List of colonies of *Anoteropora latirostris* used in this study.

| **Sample number** | **Colonies** | **Station** | **Used for** |
| --- | --- | --- | --- |
| **SMF 60000** | 1 | 283 KU | Macro images (Fig. 1(a)–(c)) |
| **SMF 60001** | 1 | 283 KU | SEM (Fig. 1(d)) |
| **SMF 60002** | 1 | 283 KU | Micro-CT (Fig. 2(a)–(b)) |
| **SMF 60003** | 1 (sectioned) | 283 KU | EBSD, Micro-CT, (Fig. 3(a)–(e), Fig. 6) |
| **SMF 60004** | 1 (fragment) | 236 KD | SEM (Fig. 4(a)–(d)) |
| **SMF 60005** | 1 (sectioned) | 283 KU | SEM, NanoSIMS (Fig. 5a-f, Suppl. Fig. S5(a)–(f)) |
| **SMF 60006** | 3 | 283 KU | SEM (unfigured) |
| **SMF 60007** | 1 | 283 KU | SEM, BSE (Fig. S2) |
| **SMF 60008** | 1 | 283 KU | SEM (unfigured) |
| **SMF 60009** | 1 | 283 KU | SEM (unfigured) |
| **SMF 60010** | 3 (fragments) | 283 KU | SEM (unfigured) |
| **SMF 60011** | 1 | 236 KD | Macro images (unfigured) |
| **SMF 60012** | 1 | 236 KD | Prestudy |
| **SMF 60013** | 3 | 283 KU | Prestudy |
| **SMF 60014** | 33 fragments | 283 KU | Prestudy |
| **SMF 60015** | 5 fragments | 283 KU | Prestudy |
| **SMF 60016** | 79 fragments | 283 KU | Prestudy |
| **SMF 60017** | 43 fragments | 283 KU | Prestudy |
| **SMF 60018** | 45 fragments | 236 KD | Prestudy |
| **SMF 60019** | 1 | 236 KD | Prestudy, SEM |
| **ZSM 20190252** | 3 (2 fragmented) | 283 KU | SEM (Suppl-fig. S3 (a)-(h)) |
| **ZSM 20190253** | 10 | 283 KU | Prestudy |

**Supplementary Table S2.** Measured CN^–^/C^–^ and Si^–^/C^–^ ratios for four sample areas and selected sub-regions (denoted by A, B and C for the respective fields, see Figs. 5 and S6). All errors are 1 sigma. Note the ratios for the epoxy references are distinct from those for all maps across the aragonite-calcite boundary indicating that the analyses are free of epoxy contamination. Only exception is B_a_01#C, which is identical to epoxy and was therefore excluded from the discussion.

|  | **^12^C^14^N^–^/^12^C^–^** | **err** | **^28^Si^–^/^12^C^–#^ (×10^–3^)** | **err (×10^–3^)** |
| --- | --- | --- | --- | --- |
| B_a_01#A | **2.31** | **0.01** | **4.11** | **0.11** |
| B_a_01#B | **1.48** | **0.01** | **3.20** | **0.23** |
| B_a_01#C | **0.70** | **0.01** | **6.21** | **0.28** |
| B_a_02 | **1.56** | **0.01** | **9.01** | **0.34** |
| B_a_03#A | **4.14** | **0.01** | **6.15** | **0.11** |
| B_a_03#B | **2.10** | **0.01** | **10.24** | **0.23** |
| B_b_1 | **6.27** | **0.02** | **13.20** | **0.28** |
| Epoxy_reference_1 | **0.68** | **0.0003** | **0.062** | **0.003** |
| Epoxy_reference_2 | **0.53** | **0.0002** | **0.035** | **0.001** |

**^#^**For SiC, the respective ion ratios are typically >3×10^–1^


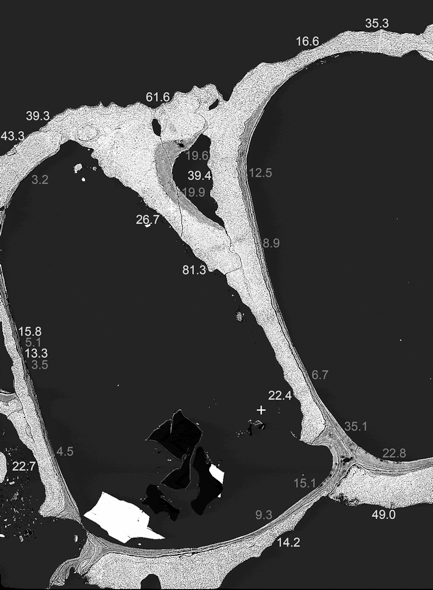


**Supplementary Fig. S1:** BSE image of a sectioned and polished colony of *Anoteropora latirostris* (SMF 60003) with measurements of wall thicknesses for calcitic (dark grey) and aragonitic layers (light grey). Area analysed is the same as the one mapped by EBSD, for location see Fig. 3c (dashed outline).


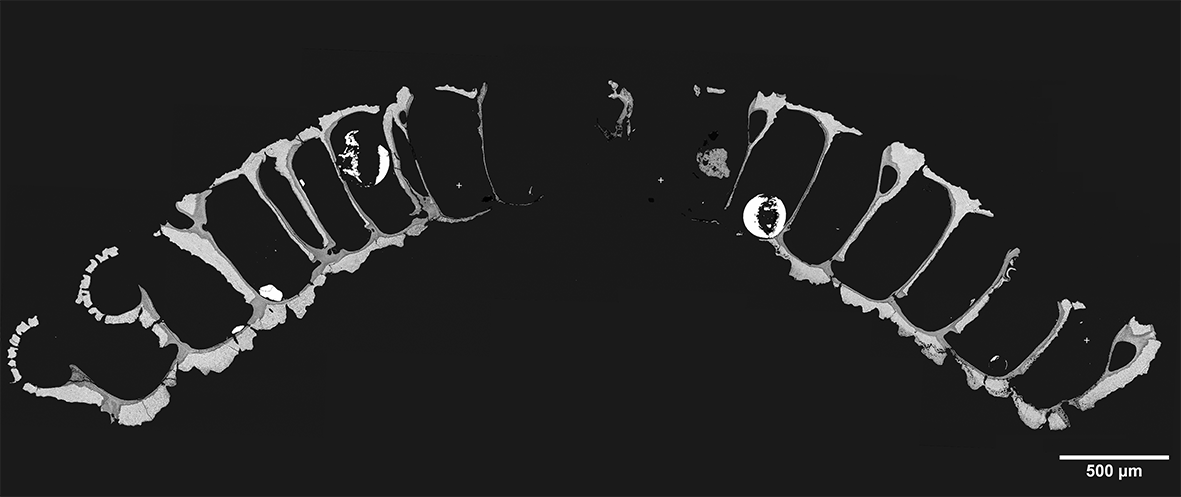


**Supplementary Fig. S2:** Backscatter Electron image of polished section of sample SMF60007. Note the distribution of aragonite (light grey) consistently on the distal side of the lateral walls. Calcite in dark grey. Ancestrula area in this colony is not preserved in this section through the centre.


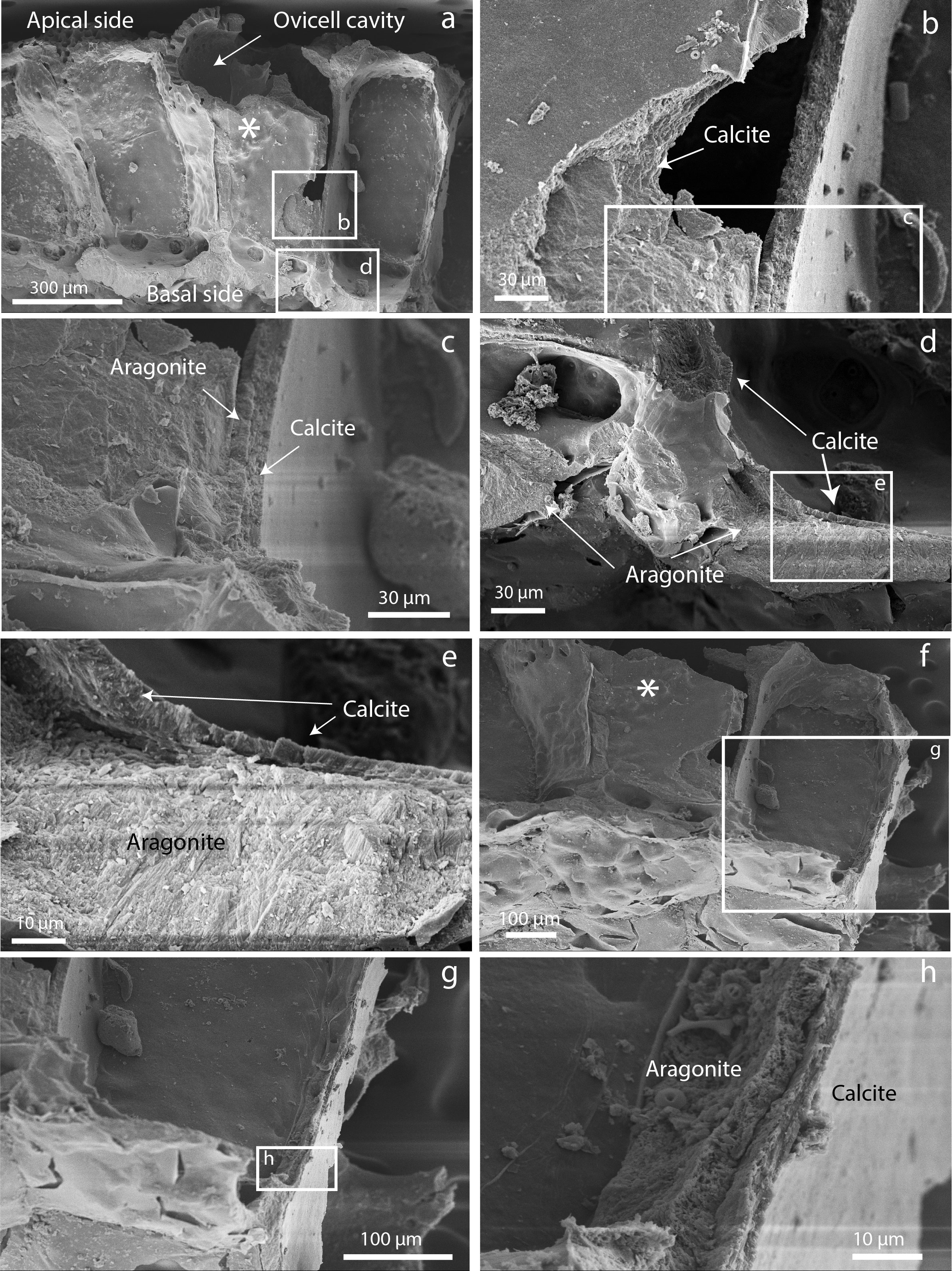


**Supplementary Fig. S3:** SEM images showing fractured walls defining the chambers of individual autozooids (Sample ZSM20190252). **(a)** Broken piece of colony showing several zooid chambers and their delineating skeletal walls. The same area is depicted at a slightly different angle in (f) – refer to asterisk for orientation. Note that the fracture surface runs in steps towards the reader relative to the imaged surface. In this way, the autozooid chamber on the right in (a) reveals the bottom-wall in addition to the fractured lateral walls, while the adjacent chamber to the left shows the top-wall of this chamber (towards the reader) in the image plane. **(b)** Close-up of area marked by white rectangle ‘b’ in (a) showing the fractured top-wall revealing the typical platy morphology of the calcitic top-wall. **(c)** Close-up of the lateral wall in (b) marked with rectangle ‘c’ shows the distal side of the wall consisting of aragonite. **(d)** Close-up of the basal area of the autozooid chamber marked with rectangle ‘d’ in (a) showing the innermost platy calcite wall, overlain by fibrous aragonite towards the outside of the colony. **(e)** Further magnification of area marked with rectangle ‘e’ in (d). **(f)** overview image of the fractured wall around the autozooid chamber depicted in (a) focussing on the lateral wall on the right. **(g)** Close-up of area marked by rectangle ‘g’ in (f), and **(h)** further magnification of area in rectangle ‘h’ in (g) showing the bimineralic lateral wall with the distal part of the wall consisting of aragonite, in analogy to the lateral wall architecture shown in (c).


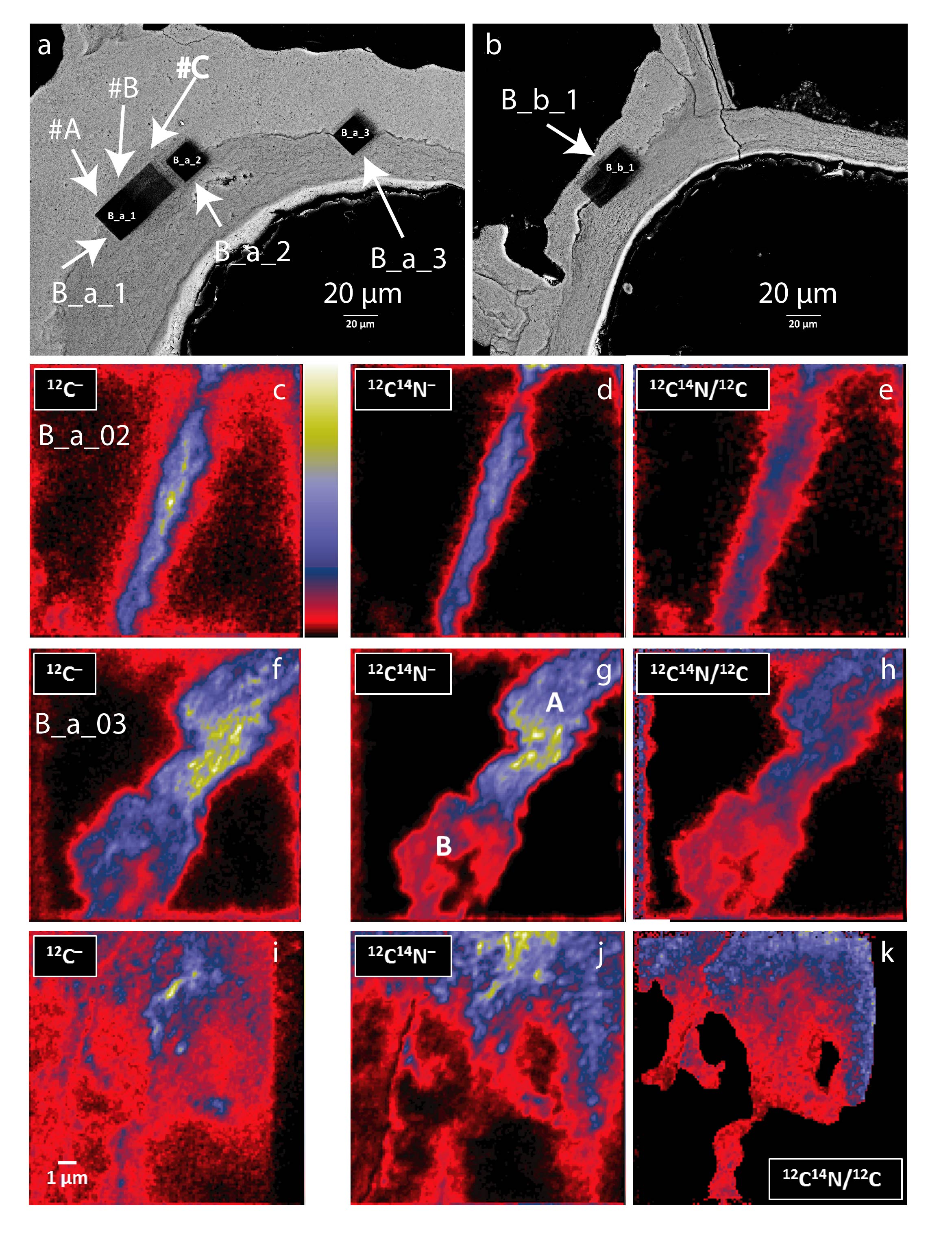


**Supplementary Fig. S4:** BSE images and NanoSIMS maps of the sectioned and polished specimen (SMF60005) in epoxy (Fig. 5a) **(a)** close-up of the areas B_a_2 and B_a_3 mapped by NanoSIMS after analysis (for area B_a_1 see Fig. 5) at the boundary between calcite (dark grey) and aragonite (light grey). **(b)** close-up of area B_b_1 (for general locations see Fig. 5). NanoSIMS analysed areas show up as dark grey squares of 15x15 µm^2^ each. NanoSIMS maps (mirrored) for carbon **(c)**, nitrogen **(d)** and calculated carbon/nitrogen ratios **(e)** for area B_a_2. (f) to (k) show respective maps for areas B_a_3 and B_b_1. Colour scale next to (c) indicates signal intensities.


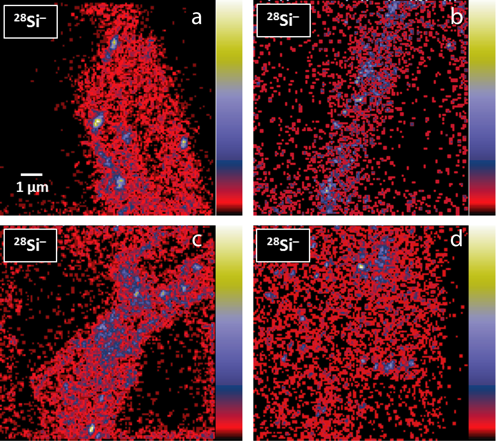


**Supplementary Fig. S5:** NanoSIMS maps (mirrored) of ^28^Si^-^/^12^C^-^ for areas B_a_01 **(a)**, B_a_02 **(b)**, B_a_02 **(c)** and B_b_1 **(d)** showing very low concentrations in each of these maps, attesting to the absence of contamination during surface polishing of the area.

**Supplementary Fig. S6:** (separate file) Crystallographic orientation map by Electron Backscatter Diffraction in original resolution (cf. downsampled version in Fig. 6c). Red grain boundaries are twin planes between aragonite grains.


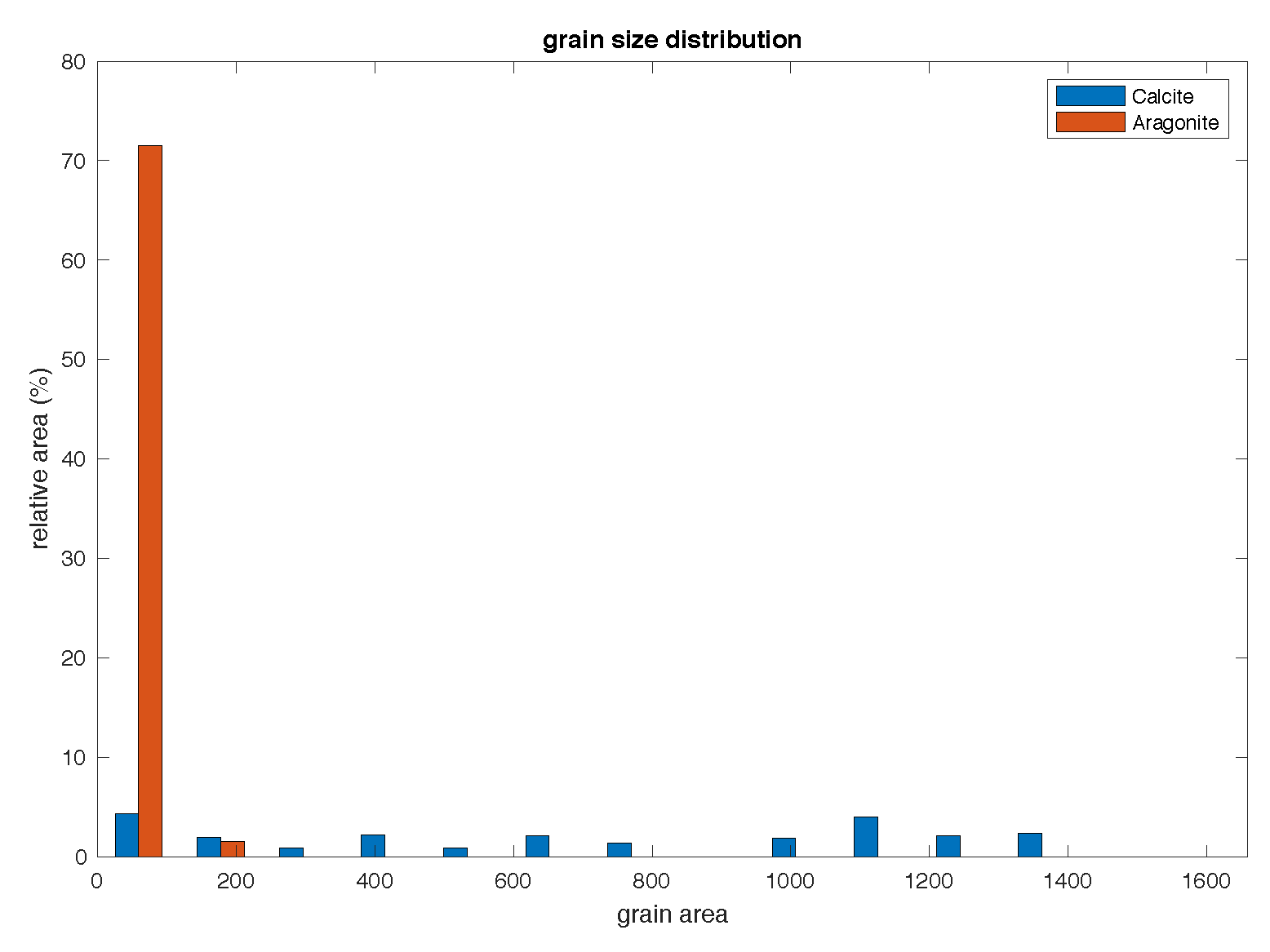


**Supplementary Fig. S7:** Grain sizes in micrometer of calcite (blue) and aragonite (red) grains for the segment mapped by EBSD. Note the variable and large sizes of calcites versus the mostly small-sized aragonite grains.


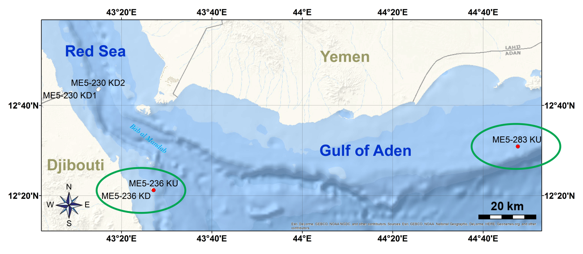


**Supplementary Fig. S8:** Map of the sampling stations during RV *Meteor* expedition 5/2 “Mindik” in March 1987 in the Gulf of Aden with the sampling stations of material used for this study indicated as red dots inside green ellipses. Map modified after template map by M. Sonnewald.

**Supplementary Methods**

Sample collection in March 1987 during RV *Meteor* expedition 5/2 “Mindik” from dredged sediment in the Gulf of Aden. Focus of this cruise was the investigation of deep-sea fauna in the aphotic zone of the Red Sea and the two stations where bryozoans were collected are the only two in shallow waters. The general depth range covered was approximately 300 to 2300 m. Shallow banks at 40-80 m depth were trawled on only two occasions in order to provide comparative data to understand the differences between shallow and deep waters of the region, and to investigate whether there were any shallow water immigrants to the deep Red Sea. These two trawls were those that contained *Anoteropora latirostris* materials.

The Gulf of Aden areas investigated were characterized by low standing stocks of plankton but high production rates, as it is typical for subtropical and tropical oceanic regions.

The official report on cruise Me5/2 “Mindik” does not provide any information on water parameters such as temperature. The hydrographic bottle data obtained in the Gulf of Aden during the cruise do not provide any data on the two shallow water stations, but focus on stations situated in the aphotic zone (Verch et al. 1989). A site situated near to station Me5-283 KU recorded at 77 m depth a temperature of 24.54 °C, and the salinity 36.194 ppt. (Ďuriš  2007).

Stations and sampling:

Me5-283 Ku

Indian Ocean: Gulf of Aden, interior part of the Gulf of Aden, (12° 30.9' N 44° 47.7' E - 12°31.2' N 44° 48.4' E)

76 m water depth, 16.3.1987, 11:52 - 12:12, Beam Trawl

Me5-236 KD

Indian Ocean: Gulf of Aden, Bab-el-Mandeb, (12° 21.4' N 43° 26.9' E - 12°20.6' N 43°27.3'E)

45 m water depth, 6.3.1987, 13:30 - 13:45, Dredge Trawl

Two types of gear were used for sampling at these stations: a beam trawl with a two meter frame and a net of 1 cm minimum mesh size in the cod end, (Me5-283 Ku) and a hard bottom dredge in which the net of 1 cm mesh size was protected by a chain-sack (Me5-236 KD). The gear was not deployed unless a suitable smooth bottom profile of an appropriate dredging distance was found. All sieve residues were preserved with 4% formalin on board. Upon arrival in Frankfurt am Main (Senckenberg Institute), the samples were watered and transferred to 70% Ethanol, and dried later for sorting smaller macrobenthos.

**References:**

Ďuriš, Z. (2007) New occurence of *Vercoia socotrana* Ďuriš, 1992 (Crustacea, Decapoda, Crangonidae) in the Gulf of Aden, Western Indian Ocean. - – Senckenbergiana Maritima, 37, 1–4

Nelle, W., Bettac, W., Roether, W., Schnack, D., Thiel, H., Weikert, H., Zeitzschel, B. (eds.): Meteor-Berichte 96-1 Mindik Reise Nr. 5, 2.Januar 1987-24.September 1987 Band I. Leitstelle Meteor, Institut für Meereskunde der Universität Hamburg 1996. 275 pages

Verch, N., Petzold, M., Mahnke, P., Quadfasel, D. (1989), Hydrographic bottle data obtained in the Red Sea and Gulf of Aden during RV METEOR cruise 5 -MINDIK 1987. Technical Report 2-89. Institut für Meereskunde der Universität Hamburg
