# Supplementary figures and images for: Architecture of Anoteropora latirostris (Bryozoa, Cheilostomata) and implications for their biomineralization
Source: Sci Rep. 2019 Aug 7;9:11439. doi: 10.1038/s41598-019-47848-4 (PMC6685955; doi:10.1038/s41598-019-47848-4)

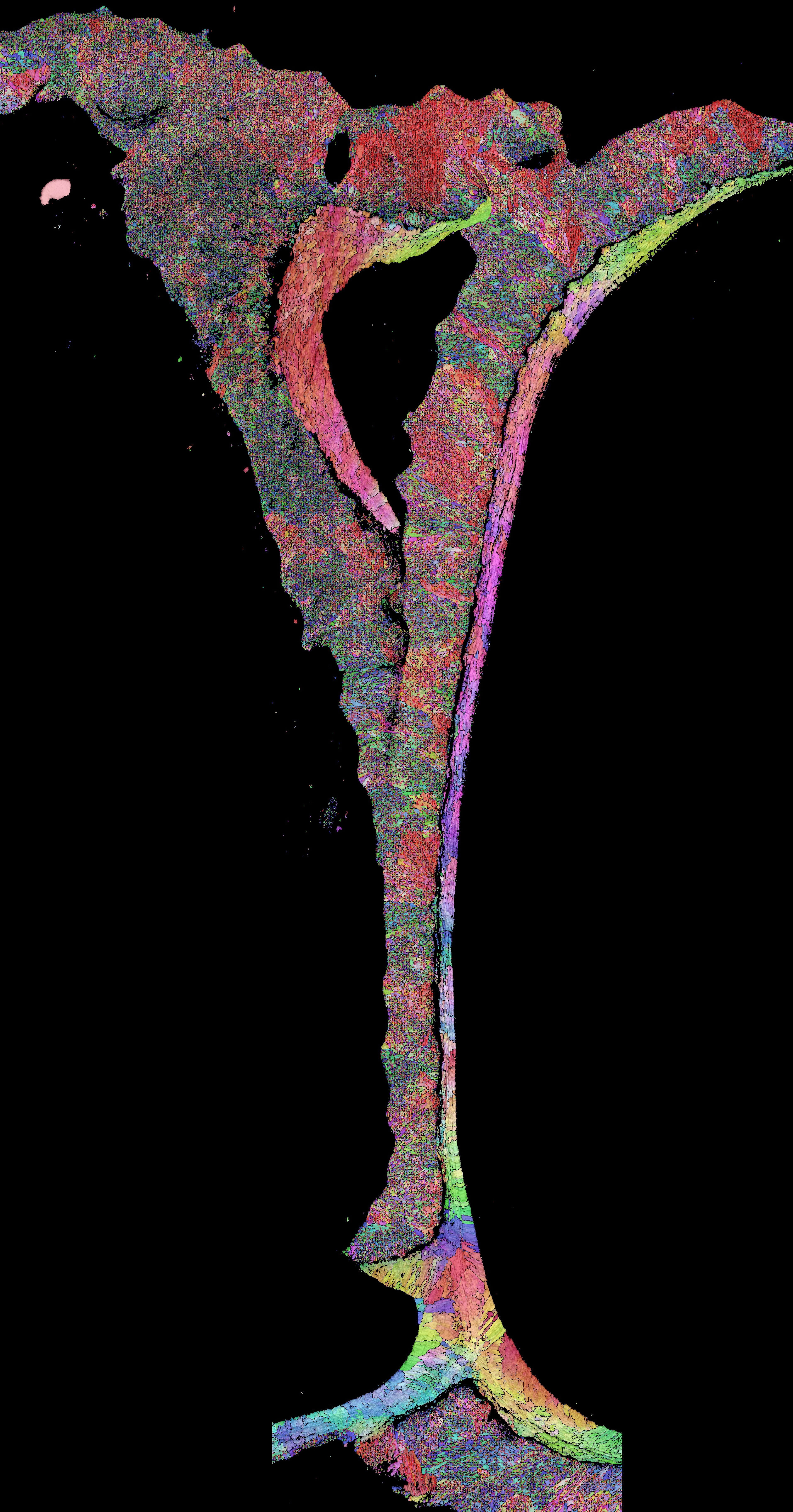

Supplement: Supplementary file 3 — Dataset 1 [file 41598_2019_47848_MOESM3_ESM.zip › Images/Anoteropora EBSD map high res.jpg]
